# Endogenous salicylic acid suppresses de novo root regeneration from leaf explants

**DOI:** 10.1371/journal.pgen.1010636

**Published:** 2023-03-01

**Authors:** Sorrel Tran, Madalene Ison, Nathália Cássia Ferreira Dias, Maria Andrea Ortega, Yun-Fan Stephanie Chen, Alan Peper, Lanxi Hu, Dawei Xu, Khadijeh Mozaffari, Paul M. Severns, Yao Yao, Chung-Jui Tsai, Paulo José Pereira Lima Teixeira, Li Yang

**Affiliations:** 1 Department of Plant Pathology, College of Agricultural & Environmental Sciences, University of Georgia, Athens, Georgia, United States of America; 2 Department of Biology, "Luiz de Queiroz" College of Agriculture, University of São Paulo, Sao Paulo, Brazil; 3 Warnell School of Forestry and Natural Resources, University of Georgia, Athens, Georgia, United States of America; 4 Department of Genetics, Franklin College of Arts and Sciences, University of Georgia, Athens, Georgia, United States of America; 5 Department of Plant Biology, Franklin College of Arts and Sciences, University of Georgia, Athens, Georgia, United States of America; 6 Department of Animal and Diary Sciences, College of Agricultural & Environmental Sciences, University of Georgia, Georgia, United States of America; Max Planck Institute of Molecular Plant Physiology: Max-Planck-Institut fur molekulare Pflanzenphysiologie, GERMANY

## Abstract

Plants can regenerate new organs from damaged or detached tissues. In the process of *de novo* root regeneration (DNRR), adventitious roots are frequently formed from the wound site on a detached leaf. Salicylic acid (SA) is a key phytohormone regulating plant defenses and stress responses. The role of SA and its acting mechanisms during *de novo* organogenesis is still unclear. Here, we found that endogenous SA inhibited the adventitious root formation after cutting. Free SA rapidly accumulated at the wound site, which was accompanied by an activation of SA response. SA receptors NPR3 and NPR4, but not NPR1, were required for DNRR. Wounding-elevated SA compromised the expression of AUX1, and subsequent transport of auxin to the wound site. A mutation in AUX1 abolished the enhanced DNRR in low SA mutants. Our work elucidates a role of SA in regulating DNRR and suggests a potential link between biotic stress and tissue regeneration.

## Author summary

Tissue regeneration is a core technology for modern agriculture and horticulture. It is widely used for crop improvement, propagation of valuable varieties and generation of chimeric plants. Plants must integrate physiological and environmental cues to complete this dramatic and sophisticated reprogramming process. Difficulties in regenerating adventitious roots from cuttings, such as the age-dependent decline of rooting, is still a bottleneck in propagating economically and ecologically important plants. We discovered that Salicylic acid (SA), a key hormone for plant defense, suppresses root regeneration from cuttings. Depleting endogenous SA or disrupting SA signaling enhances plants’ regeneration ability. Our study provides new knowledge for overcoming challenges in vegetative propagation by manipulating the SA response.

## Introduction

Plants have a remarkable ability to regenerate after wounding [1,2]. Regeneration of adventitious roots from leaf explants or stem cuts lays a foundation to propagate valuable crops and fruits for agriculture and horticulture [3]. Regeneration requires a signaling cascade from the perception of wound signals, gain of reprogramming competence, conversion of cell fate, and eventually, patterning of the new organ [4,5]. Arabidopsis leaf explants detached from a stem can develop adventitious roots from the wound site without exogenous supplementation of phytohormones [5,6]. This process is referred as *de novo* root regeneration (hereafter DNRR) [4,5].

The process of DNRR solely relies on the dynamic interactions among endogenous hormones, since no exogenous hormones (e.g., auxin or cytokinin) are added to induce cell differentiation [7]. In the current model of DNRR, jasmonic acids (JAs) serve as a wound-induced early signal to activate a group of transcription factors, including the ETHYLENE RESPONSE FACTOR 109 (ERF109) shortly after cutting [8,9]. ERF109 serves as a link between early JA signals and subsequent auxin biosynthesis because it directly activates the expression of *ANTHRANILATE SYNTHASE α1* (*ASA1*)—a tryptophan biosynthesis gene in the auxin production pathway [8]. In addition to *ASA1*, other genes for auxin biosynthesis (e.g., *YUCCA1*, *YUCCA4*, and *YUCCA6*) are activated in distal tissues, leading to a synthesis of auxin in the leaf mesophyll cells [8]. Newly synthesized auxin is transported to the wound site where it activates members of the auxin response transcription factors (ARFs). The activation of auxin response is evident one day after cutting (DAC) at the wound site [5]. ARF7 and ARF19 can directly activate *WUSCHEL RELATED HOMEOBOX 11* and *12* (*WOX11* and *12*) to initiate cell fate transition [5]. Auxin-induced expression of *WOX11* in cambium cells is considered as a first step for cell fate transition during DNRR [10, 11], which occurs approximately at two DAC. WOX11/12 subsequently activates a root quiescent center marker, *WOX5*, initiating adventitious root formation [10].

Salicylic acid (SA) is essential to launch a robust defense against various biotrophic and hemi-biotrophic pathogens such as *Pseudomonas syringae* pv. *tomato* DC3000 [12]. In Arabidopsis, mutants defective in SA biosynthesis or signaling show enhanced susceptibility to viral, bacterial, oomycete, and fungal pathogens [13]. SA is also involved in responses to abiotic stresses, such as drought, and in the regulation of development, including flowering time and root patterning [14–16]. The current understanding of the SA signaling pathway is largely gained from studies of plant immunity. SA is perceived by paralogs of the *NONEXPRESSER OF PATHOGENESIS RELATED 1 (NPR1)* gene. Six Arabidopsis *NPR1* paralogs (*NPR1*, *NPR2*, *NPR3*, *NPR4*, *NPR5*, and *NPR6*) share a BTB/POZ (Broad-complex, Tramtrack, and Bric-a-brac/Poxvirus and Zinc-finger) domain, and an ankyrin repeat domain [17]. NPR1, NPR3 and NPR4 have been demonstrated to bind SA and to transduce SA-induced immune signaling in Arabidopsis [18–21]. NPR1 contains a transcription co-activation domain at its C-terminus and can activate the expression of genes required for Systemic Acquired Resistance (SAR) upon SA perception [22]. On the other hand, NPR3 and NPR4 act redundantly to repress SA-mediated defense responses in the absence of SA [23, 24]. NPR3 and NPR4 negatively regulate defenses by independently regulating NPR1-controlled genes [20] or through degrading NPR1 [19].

Here we report that endogenous SA suppresses DNRR from leaf explants. SA response is activated rapidly after cutting accompanied by an accumulation of free SA. NPR4 serves as a key receptor of SA in regulating the suppression of DNRR, and distinct signaling components are recruited for SA-mediated defense and regeneration. SA inhibited the transport of auxin to the cutting site. AUX1 is transcriptionally suppressed by SA after cutting and its mutation rescues the enhanced rooting in an SA-deficient mutant. Taken together, our results revealed key signaling components required for the SA-mediated suppression of wound-induced DNRR.

## Results

### Endogenous SA suppressed DNRR from Arabidopsis leaf explants

**Fig 1:**
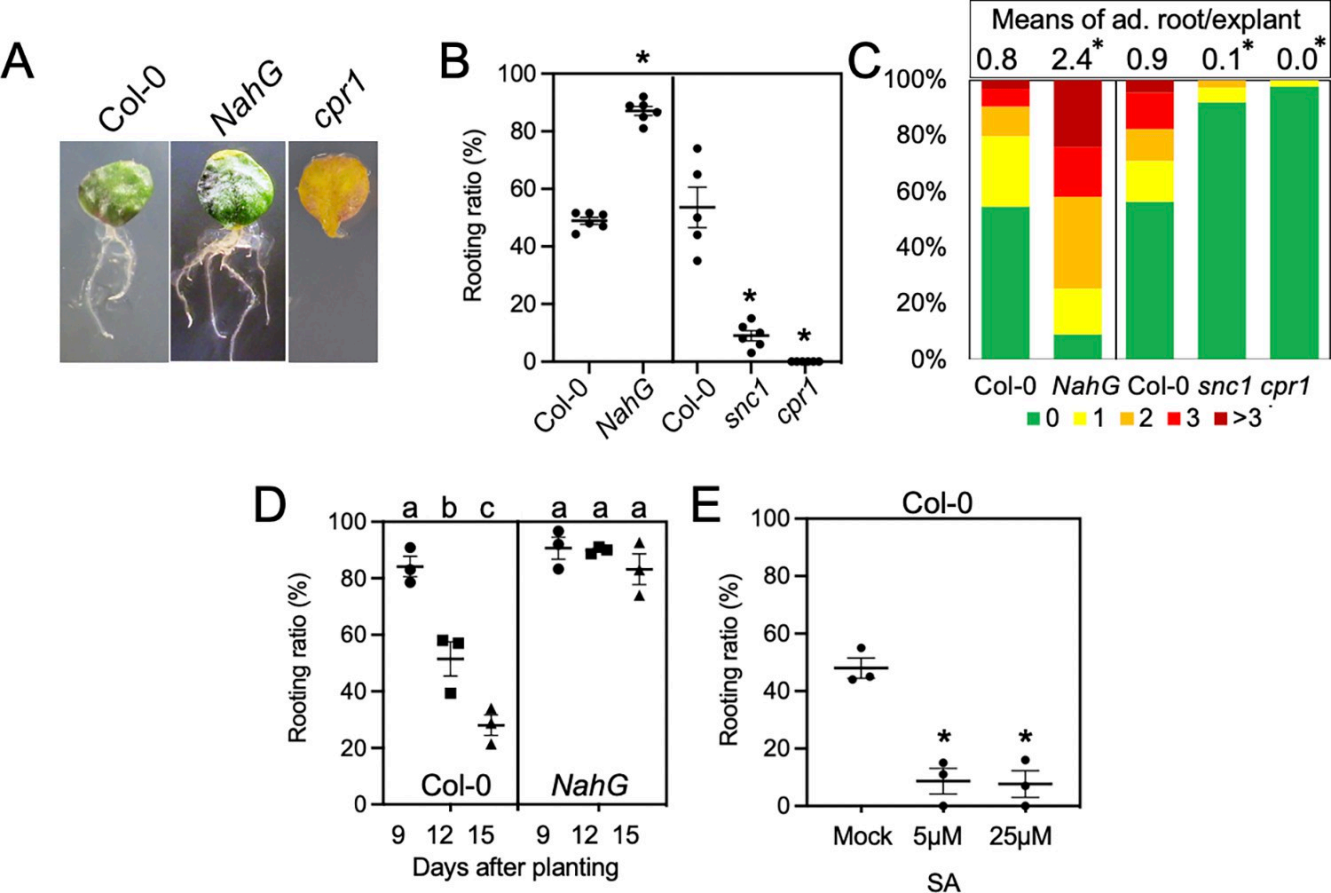
SA repressed the formation of adventitious roots from leaf explants. (A) Representative images of leaf explants at 11 DAC from wild type and mutants. Explants were cut from the first two rosette leaves. (B) The rooting ratio in wild type Col-0 and mutants. * indicates p<0.01 when compared to Col-0 using student *t*-test. Long and short bars represent means and standard errors, respectively. (C) The proportion of leaf explants with indicated number of adventitious roots from wild type and mutants. * indicates significant difference using a Mann–Whitney U test when compared to Col-0. (D) The rooting ratio of explants from plants with different age. Different letters indicate significant difference using one way ANOVA. (E) Rooting ratio of Col-0 explants with SA treatment. Each dot in B, D, and E represents an independent experiment with 40–60 explants. Error bars indicate standard error. The rooting data were collected at 11 DAC.

To investigate the role of endogenous SA in DNRR, we compared the rooting ability of leaf explants from the wild type, Columbia-0 (Col-0), and transgenic plants overexpressing *NahG*, a salicylate hydroxylase derived from the bacterium *Pseudomonas putida*, under a constitutive (cauliflower mosaic virus 35S, hereafter 35S) promoter (*35S*::*NahG*, hereafter NahG). Overexpression of NahG reduces the free SA level by converting SA to catechol [25]. The ratio of leaf explants forming adventitious roots were significantly higher in NahG than those in wild type, Col-0 (Fig 1A and 1B). NahG explants also generated an increased number of adventitious roots on each explant (Fig 1C). We also observed a reduced rooting ability in older Col-0 leaf explants, which is consistent with a previous work that showed an age-dependent decline in regeneration [26,27] (Fig 1D). The age-dependent decline of rooting was completely abolished in NahG explants (Fig 1D). Conversely, in Arabidopsis mutants with a high level of SA, such as *snc1* (*SUPPRESSOR OF NPR1-1*) and *cpr1* (*CONSTITUTIVE EXPRESSER OF PR GENES 1*), the formation of adventitious roots from leaf explants was significantly suppressed (Fig 1A–1C). We acknowledge that SNC1 is involved in a microRNA pathway and immune signaling [28–30], whereas CPR1 regulates SNC1 stability, with SA elevation as an indirect effect [31]. We therefore sprayed SA onto leaves one hour before cutting to investigate an SA-specific response on DNRR. We observed that exogenous SA at a concentration beyond 5 μM inhibited adventitious root formation like the phenotypes seen in the *snc1* and *cpr1* mutants (Figs 1E and S1). Thus, we conclude that endogenous SA inhibits adventitious root formation on leaf explants.

### The SA pathway was activated after leaf excision

**Fig 2:**
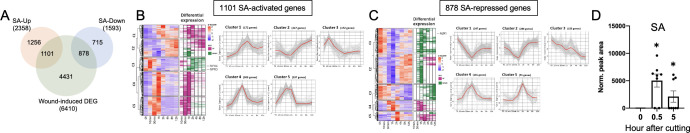
SA response was activated after wounding. (A) Venn diagram showing the overlap of genes that were differentially expressed upon wounding in the first 12 hours after cutting [8] and markers of the SA response [32]. The SA-induced genes are shown in the light pink circle, SA-repressed genes are shown in the light blue circle, and in the green circle are genes that showed altered expression upon wounding in at least one time-point of the experiment. A total of 1101 genes are activated by SA and differentially expressed upon wounding. Furthermore, 878 of the SA-repressed genes are differentially expressed in wounded leaves. (B) Expression profile of the 1101 SA-activated genes that were differentially expressed in leaves during DNRR Marker genes of the SA response were defined by the treatment of Arabidopsis seedlings with exogenous SA [32]. Genes activated by SA and differentially expressed in our experiment were submitted to hierarchical clustering based on their expression profile in wounded leaves. Differential expression at each time point is indicated with a color code (induced: purple; repressed: green). *NPR4* and *NPR3* are part of cluster 3 (C3) and are highlighted next to the heatmap. A representative profile of each cluster is shown on the right (red line: average behavior; grey line: individual genes). (C) Expression profile of the 878 SA-repressed genes that were differentially expressed in leaves during DNRR. Marker genes of the SA response were defined by the treatment of Arabidopsis seedlings with exogenous SA [32]. Genes repressed by SA and differentially expressed in our experiment were submitted to hierarchical clustering based on their expression profile in wounded leaves. Differential expression at each time point is indicated with a color code (induced: purple; repressed: green). *AUX1* is part of cluster 1 (C1) and is highlighted next to the heatmap. A representative profile of each cluster is shown on the right (red line: average behavior; grey line: individual genes). (D) Accumulation of free SA in explants. Each dot represents an independent SA-measurement from two leaf explants by LC-MS (n = 8). The Y axis is peak area normalized for loading based on internal standards.

To monitor the dynamics of the SA response during DNRR at the transcriptome level, we further investigated the expression pattern of SA-responsive genes in the first 12 hours after leaf excision [8]. SA-responsive genes were defined in Yang *et al* [32]. Similar to the present study, Yang *et al*. also used two-week-old seedlings. In total, 6410 genes were differentially expressed in at least one time point after wounding compared to the control condition (time 0). Of the 2357 SA-activated genes [32], 1101 were differentially expressed in at least one time point after cutting. Similarly, 878 of the 1593 genes that are repressed by SA were also differentially expressed in at least one time point after cutting (Fig 2A). Thus, SA-responsive genes account for 31% (1979 of 6410) of the total genes that respond to wounding within the first 12 hours.

A hierarchical clustering analysis of the SA-responsive genes showed that a subset of them were either activated or repressed within as early as 10 minutes after leaf excision, indicating a rapid SA response after cutting (Fig 2B and 2C and S1 Table). Despite the activation of SA pathway as a whole, key genes involved in NPR-mediated defense signaling were downregulated in this early wound response, implying negative feedback or a specific repression of SA-mediated defense signaling (S2A Fig). A subset of SA-repressed genes (cluster 4 and 5 in Fig 2C) was activated upon wounding, indicating potential cross-regulation by other stimuli. Indeed, 50% of all genes included in either clusters 4 and 5 can also be activated by jasmonic acid (JA) [32] (S2B Fig), a known antagonist of the SA-mediated defense gene expression [33]. Given the activation of the JA pathway after leaf excision [8], these genes in clusters 4 and 5 (Fig 2C) may represent the sector of JA-SA crosstalk in the early stage of DNRR. We also examined the levels of free SA in wounded and non-wounded leaf explants from 12-day-old seedlings grown on plates (Fig 2D). Consistent with the observed activation of the SA response, accumulation of free SA was observed 30 min after cutting (Fig 2D). Collectively, this data shows that leaf excision triggers SA accumulation and an associated transcriptional response that is highly reminiscent of the response observed after exogenous SA treatment.

### SA-mediated DNRR required distinct components from SA-mediated immunity

**Fig 3:**
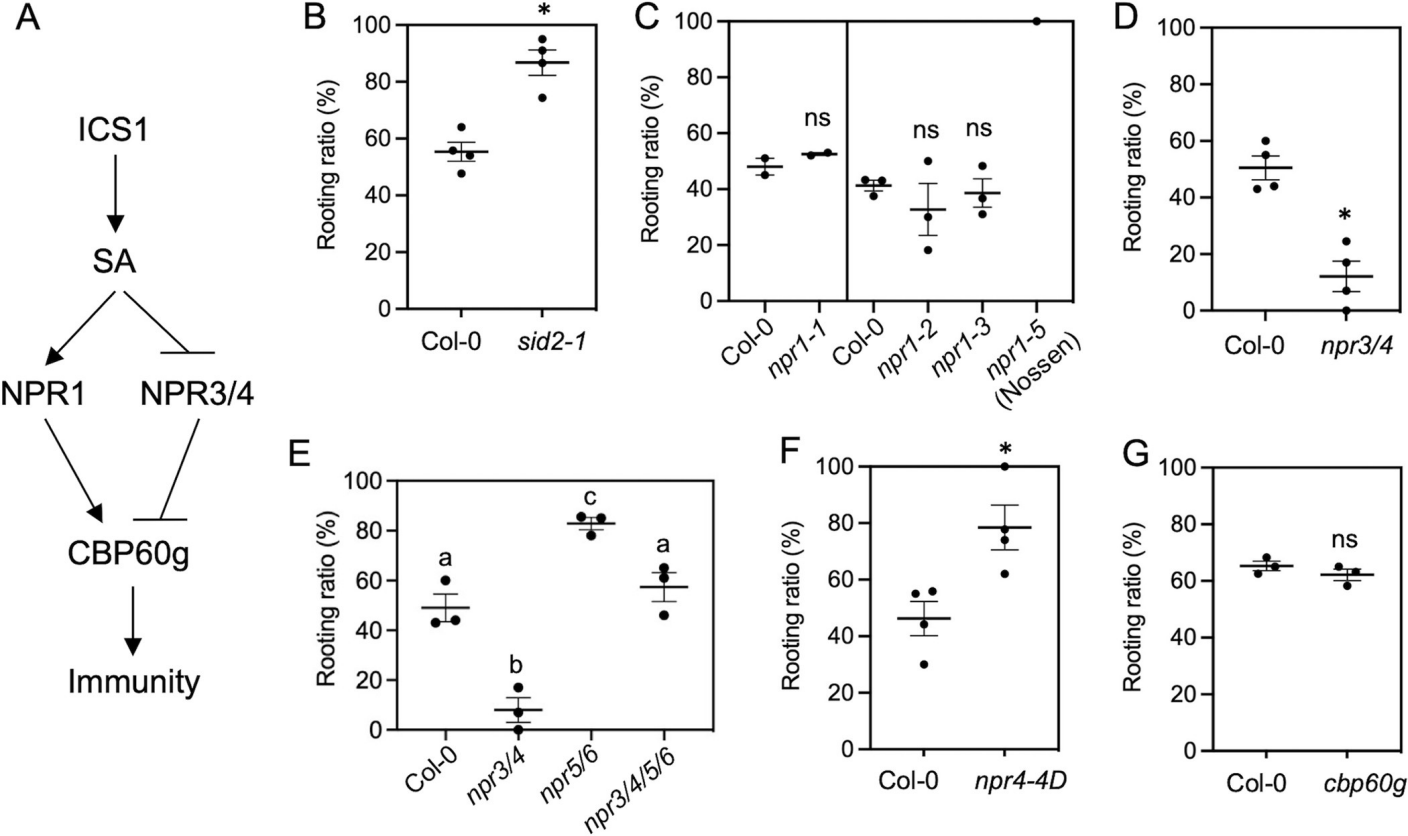
Genes involved in SA-mediated defense were differentially involved in DNRR. (A) A simplified pathway of SA signaling. (B)–(G) the rooting ratio in various SA mutants. Each dot represents an independent experiment with 40–60 explants. Error bars indicate standard error. The rooting data were collected at 11 DAC. * indicates p<0.01 when comparing to Col-0 using student t-test. ns: not significant; Different letters in (E) indicate significant difference using one way ANOVA.

SA is required for defense responses, including SAR and local defense responses against biotrophic pathogens [12]. To investigate if the same components involved in SA-mediated defense response are also involved in SA-mediated DNRR, we tested the rooting phenotype in mutants involved in SA biosynthesis (*sid2*), SA perception (*npr1*, *npr2*, *npr3* and *npr4*), and signaling (*cbp60g*) (Fig 3A).

Chloroplast localized ISOCHORISMATE SYNTHASE1 (ICS1) is required for SA biosynthesis during infection, and its mutant *sid2* (SALICYCLIC ACID INDUCTION DEFFICIENT 2) is more susceptible to bacterial and fungal pathogens [34]. Leaf explants from *sid2* mutants showed enhanced adventitious root formation (Fig 3B), indicating that ICS1 is also responsible for the biosynthesis of SA in suppressing rooting. NPR1 and NPR3/4 can activate or repress, respectively, defense gene expression after binding to SA [19–22,35]. A *npr1* mutant (*npr1-5*) was previously shown to enhance DNRR [26]. However, we found that several different alleles of *npr1* did not show altered DNRR, despite a clear compromised immune response in these alleles [36] (Fig 3C). We did not observe enhanced rooting in young (12 days after planting) or old (15 days after planting) leaves of *npr1-3* (S3A Fig). Since *npr1-5* was isolated in a Nossen (No-0) background, we compared the rooting capacity of explants from Col-0, No-0 and *npr1-5*. No difference was observed between No-0 and *npr1-5*, albeit both showed a high rooting capacity compared to Col-0 (S3B Fig). In addition, explants from F1 seedlings of a cross between *npr1-5* and *npr1-1* showed the same rooting ratio as those from the cross between *npr1-5* and Col-0, indicating that homozygosity of *NPR1* did not change DNRR (S3C Fig). Since NPR3 and NPR4 redundantly suppress defense gene expression [23], we examined the DNRR response in a *npr3-1 npr4-3* double mutant and found that adventitious root formation was dramatically suppressed (Fig 3D). Two NPR1-like proteins, NPR5 and NPR6 (also known as BLADE ON PETIOLE 2 (BOP2) and BLADE ON PETIOLE 1 (BOP1)), negatively regulate DNRR [37]. Interestingly, the quadruple mutant of *npr3/4/5/6* showed similar rooting ability as Col-0, indicating that NPR3/4 and NPR5/6 play opposite roles in regulating DNRR (Fig 3E). To further validate that the function of NPR4 in regulating DNRR is due to its role as a SA receptor, we examined the DNRR phenotype in a gain-of-function *npr4-4D* mutant. *Npr4-4D* carries an Arginine to Glutamine mutation at position 419 (R419Q), which blocks the SA binding of NPR4 [20]. We found that leaf explants from *npr4-4D* had a higher rooting ratio than Col-0 (Fig 3F), indicating that the SA binding ability of NPR4 is required for SA-mediated suppression of DNRR. Furthermore, *npr4-4D* showed reduced sensitivity to exogeneous SA in suppressing rooting (S3 Fig). *Arabidopsis thaliana Calmodulin Binding Protein 60g* (*CBP60g*) is a direct transcriptional target of NPR 3/4 [20]. The *cbp60g* mutant showed reduced disease resistance to *Pseudomonas* [38,39]. Interestingly, compared to Col-0, the rate of root initiation was not altered in *cbp60g* (Fig 3G), suggesting that SA-mediated defense and DNRR diverge upstream of CBP60g. Thus, SA biosynthesis via the isochorismate synthase (ICS) pathway are required for both SA-mediated defense and DNRR. The NPR3/4, but not the NPR1/CBP60g, immune signaling node is involved in SA-mediated DNRR.

### SA inhibits auxin transport to the cutting site during DNRR

**Fig 4:**
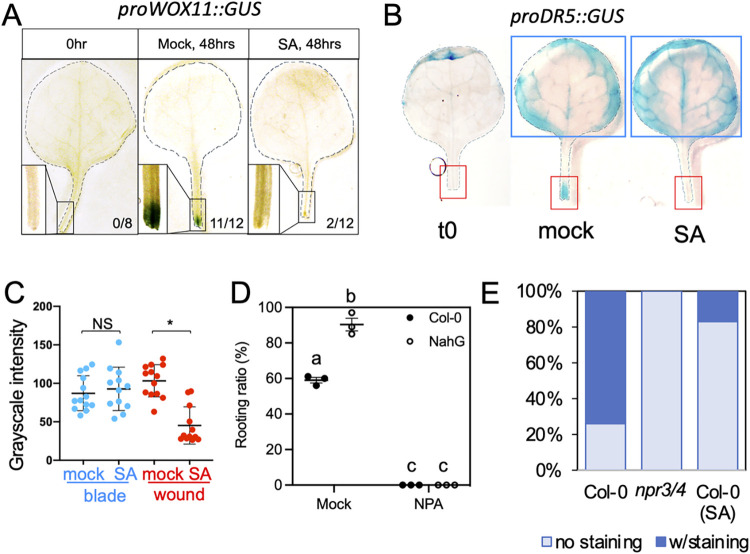
SA inhibited auxin transportation to the cutting site. (A) Expression pattern of *proWOX11*::*GUS* in explants treated with SA or mock. Note the lack of staining at the cut site of SA treated samples. Samples were stained at 2 DAC. (B) SA treatment compromised the activation of auxin reporter, *proDR5*::*GUS*, at the cut site (red box), but not in distal tissue (blue box). Cutting was made at the base of petiole to distinguish the staining of DR5 at the wound site versus distal sites. T0 sample was stained on an intact plant and cut after staining. Mock and SA samples were stained at 1 DAC. (C) Quantification of GUS staining showed that SA treatment specifically reduced staining at the wound site but not in the distal tissue. Each dot represents an individual leaf sample. *: *p*<0.01 using student *t*-test. Y axis represents gray scale intensity. BTH, an SA analogue, was used in the SA treatment (A, B and C) at a concentration of 300 μM. (D) Rooting ratio in Col-0 and NahG after NPA treatment. NPA treatment (1μM) abolished the high rooting phenotype in NahG. Different letters indicate significant difference using one way ANOVA. (E) Ratio of explants with proDR5::GUS activity at the cutting site. *npr3/4* mutation and SA treatment reduced the staining at cutting sites. Around 40 explants were analyzed for each genotype.

To investigate how SA affects DNRR, we checked the expression of *WOX11* as an indicator of cell fate transition from cambium to root founder cells under SA treatment. In mock treated explants, GUS activity driven by the *WOX11* promoter (*proWOX11*::*GUS*) was evident at two DAC only at the wound site. In contrast, in the majority of SA-treated explants (10 out of 12), *proWOX11*::*GUS* activity was not observed at the wound site (Fig 4A). Consistently, the expression of *WOX11* was reduced in the *cpr1* background containing high SA (S4A Fig). These results indicate that SA represses DNRR by regulating events upstream of *WOX11* activation.

Auxin synthesis and transport plays an essential role in DNRR, leading to the activation of *WOX11* expression [11]. We then monitored the spatial pattern of GUS activity driven by a synthetic auxin response element DR5 promoter (hereafter proDR5::GUS) [40]. When explants were treated with SA, *proDR5*::*GUS* was activated in leaf blades, but the activity at the wound site was reduced (Fig 4B and 4C). This suggests that SA did not block the auxin biosynthesis in distal tissues in response to wounding but interfered with the auxin transport to the wound site or the local auxin response. To test whether auxin transport is required for enhanced rooting in NahG plants, we treated explants from Col-0 and NahG with 1 μM of Naphthylphthalamic acid (NPA), an inhibitor of polar auxin transport. NPA completely blocked rooting in NahG as well as Col-0 (Figs 4D, S4B, and S4C), indicating that polar transport of auxin is required for enhanced DNRR in NahG. Furthermore, the DR5-driven GUS activity was dramatically decreased at the cutting sites from *npr3/4* explants as observed from Col-0 explants treated with SA (Fig 4E). These results suggest that SA suppresses wound-induced rooting by inhibiting auxin transport to the cutting site.

### SA suppressed DNRR by interfering with auxin transport

**Fig 5:**
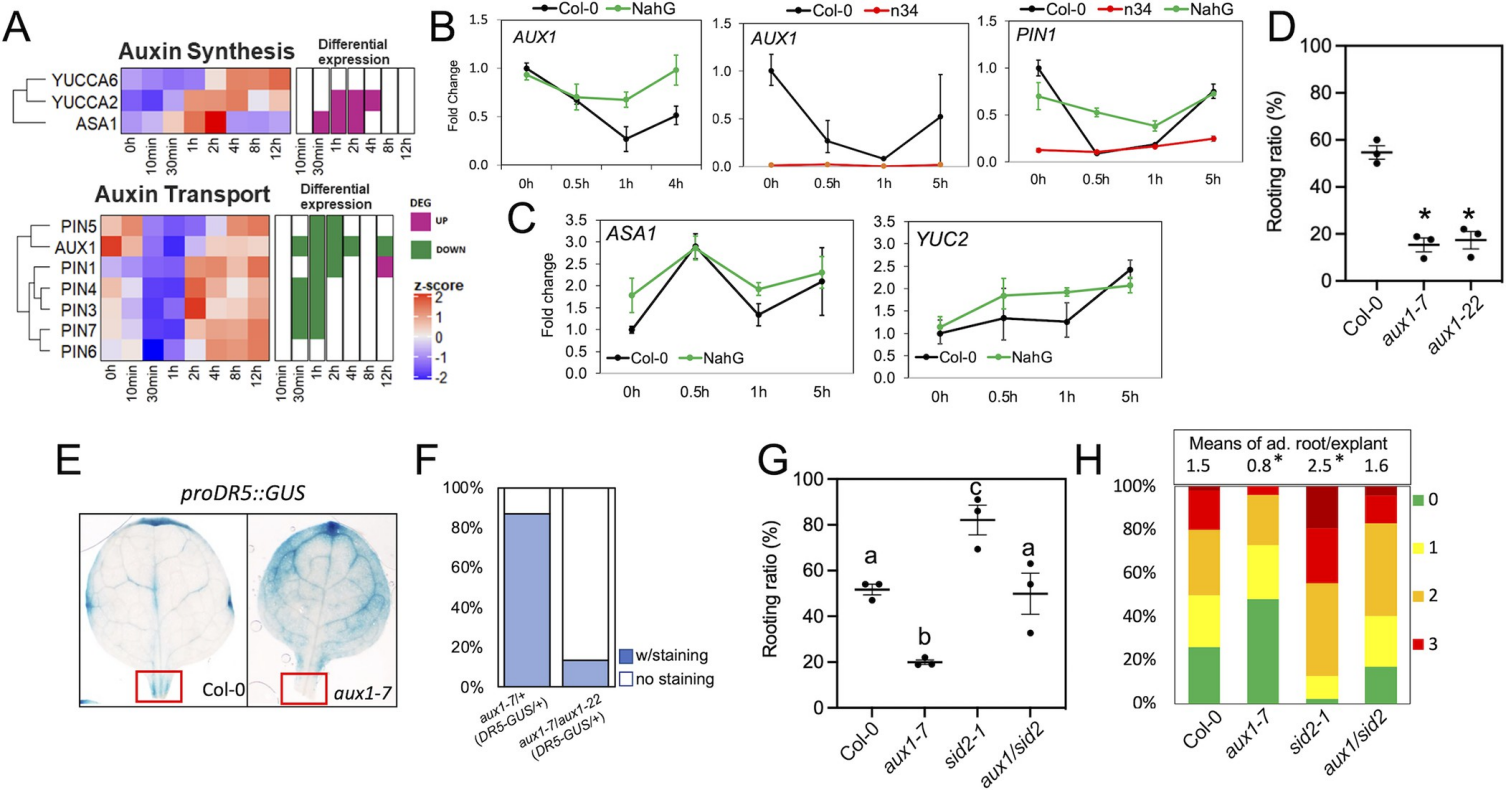
AUX1 acted downstream of SA to promote DNRR. (A) Expression pattern of genes involved in auxin biosynthesis and transport after leaf detaching. (B) and (C) Expression pattern of *AUX1*, *PIN1* (B) *and YUC2*, *ASA1* (C) after cutting in Col-0, *npr3/4* and NahG mutant. *TUB2* (AT5G62690) was used as an endogenous control in qPCR analysis. Error bars indicate standard deviation of three technical repeats. (D) Rooting ratio in loss-of-function *aux1-7* and *aux1-22*. * indicates *p*<0.01 when compared to Col-0 using student *t*-test. (E) Staining of *proDR5*::*GUS* in Col-0 and *aux1-7* mutant. Note the lack of GUS activity in the boxed cut site in *aux1-7* mutant. (F) Proportion of *proDR5*::*GUS* staining at the cut sites of explants from different crosses. *Aux1-7* mutant carries homozygous *proDR5*::*GUS*. (G) Rooting ratio in *aux1-7 sid2-1* double mutant. The *aux1-7* mutant restored the high rooting ratio of *sid2* explants to wild type levels. Different letters indicate significant difference using one-way ANOVA. (H) Average number of adventitious roots on *aux1-7 sid2-1* explants. Statistics was performed by using a Mann–Whitney U test.

Both auxin biosynthesis and transport are important for wound-induced DNRR [8,41,42]. In analyzing the early responsive genes to leaf detaching, we found that genes involved in auxin synthesis such as *ANTHRANILATE SYNTHASE α1 (ASA1)*, *YUCCA 2* and *6* (*YUC2* and *YUC6*), were upregulated as previously reported (Fig 5A) [8,41,42]. No clear pattern was observed in *AUXIN RESPONSE TRANSCRIPTION FACTOR*s (*ARF*)s (S5A Fig). However, genes for auxin transport, including *AUX1* (*AUXIN RESISTANT 1*) and *PIN-FORMED* genes (*PIN*s) were largely suppressed from 30 minutes to two hours after cutting, which coincided with the timing of the SA response (Fig 5A). We found that the down-regulation of *AUX1* and *PIN1* was compromised in NahG, but further enhanced in the *npr3/4* double mutant (Fig 5B). Auxin accumulation was not different between Col-0 and NahG (S5B Fig), and the wound-induced expression of *ASA1* and *YUC2* was not altered by NahG (Fig 5C). Taken together, endogenous SA may regulate auxin transport at the early stage after cutting (Fig 5C).

Since *pin1* mutants show pleiotropic developmental defects, we measured rooting ability in loss-of-function alleles of *aux1* to test the genetic interaction between SA signaling and auxin transport during DNRR. *aux1-7* and *aux1-22* showed reduced adventitious root formation (Fig 5D). *DR5*::*GUS* activity was significantly reduced at the cutting site in the *aux1-7* mutant (Fig 5E). However, the intensity of pro*DR5*::*GUS* in the leaf blade of *aux1* was comparable to that in Col-0, implying that wound-induced auxin biosynthesis was still activated in the *aux1* mutant. To minimize the impact of different linage history associated with the *proDR5*::*GUS* reporter in Col-0 (DR5::GUS) and *aux1-7* (DR5::GUS), we crossed *aux1-7* (DR5::GUS) to Col-0 and *aux1-22* and examined the staining pattern in their F1 explants. The explants with homozygous *aux1* (*aux1-7*/*aux1-22*, *proDR5*::*GUS*/+) showed reduced *proDR5*::*GUS* expression at the cutting site compared to those with heterozygous *aux1-7* mutation (*aux1-7*/*+*, *proDR5*::*GUS*/+) (Fig 5F).

To test the genetic interaction between AUX1 and SA signaling, we crossed *aux1-7* to a *sid2* mutant which has reduced SA levels and enhanced DNRR (Figs 3B and 5G). The *aux1 sid2* double mutant showed a rooting ratio similar to Col-0 (Fig 5G) and had a high number of adventitious roots (Fig 5H) like *sid2* explants. This suggests that AUX1 activity partially contributes to the enhanced DNRR phenotypes in SA-deficient mutants. Taken together, these results indicate that SA suppresses DNRR partially by repressing auxin transport.

## Discussion

Almost all phytohormones are mobilized during the process of wound-induced regeneration [2]. The role of SA and its analogs in *de novo* organogenesis is still under investigation. Recent studies in Arabidopsis highlights the involvement of SA in regeneration [26]. SA response was found to be activated one day after cutting and acted downstream of glutamate receptors to repress multiple forms of regeneration [26]. We observed an earlier wound-induced SA surge within 1 hour after cutting (Fig 2). It is possible that multiple waves of SA responses triggered by wounding regulate distinct stages of regeneration. In the process of DNRR, callus formation, cell fate transition, and organogenesis occur following leaf cutting [5]. For example, SA may antagonize wound-induced JA responses at an early stage and interfere with auxin-mediated root meristem patterning after root initiation [43]. Various regeneration systems using leaf or stem explants showed that SA can play positive or negative roles in organogenesis [44–51]. These contradictory observations may be caused by the application of SA at different stages of the regeneration process.

NPR proteins are key regulators of SA-mediated defense. NPR1 and NPR3/4 play opposite roles in regulating defense gene expression [20]. Our data showed that NPR3/4 promote root regeneration while NPR1 is dispensable for this process (Fig 3). Previous work showed that the *npr1-5* allele had an enhanced rooting ratio and abolished the age-dependent decline of rooting [26]. It is possible that NPR1-regulated defense and regeneration can be decoupled by mutations that disrupt the interaction between NPR1 and specific protein partners. It is also noteworthy that *npr1-5* is in the Nossen background [52,53], whereas the mutations examined in this study are in Col-0. The difference in these genetic backgrounds may explain the difference in the effects of these mutations on root regeneration [52] (S3B and S3C Fig). Alternatively, the varying DNRR phenotypes in *npr1* mutants suggest that different mutations of NPR1 may have different sensitivity to SA-mediated suppression of DNRR and immunity. Given the observation that *cbp60g* mutant did not alter DNRR, we reason that the negative role of SA on DNRR is not a secondary consequence of activating defense response. NPR5/6 lacks the key residues for SA binding and the *npr5/6* double mutant did not show altered response to bacterial pathogen and SA treatment [54,55]. NPR5/6 may antagonize NPR3/4 function by competing for common interactors or directly binding NPR3/4 rather than acting as a SA receptor. Indeed, multiple TGA transcription factors can interact with both NPR4 and NPR5/6 [23,56–58]. It will be interesting to dissect the specificity and dynamics of NPR-TGA interaction during regeneration.

**Fig 6:**
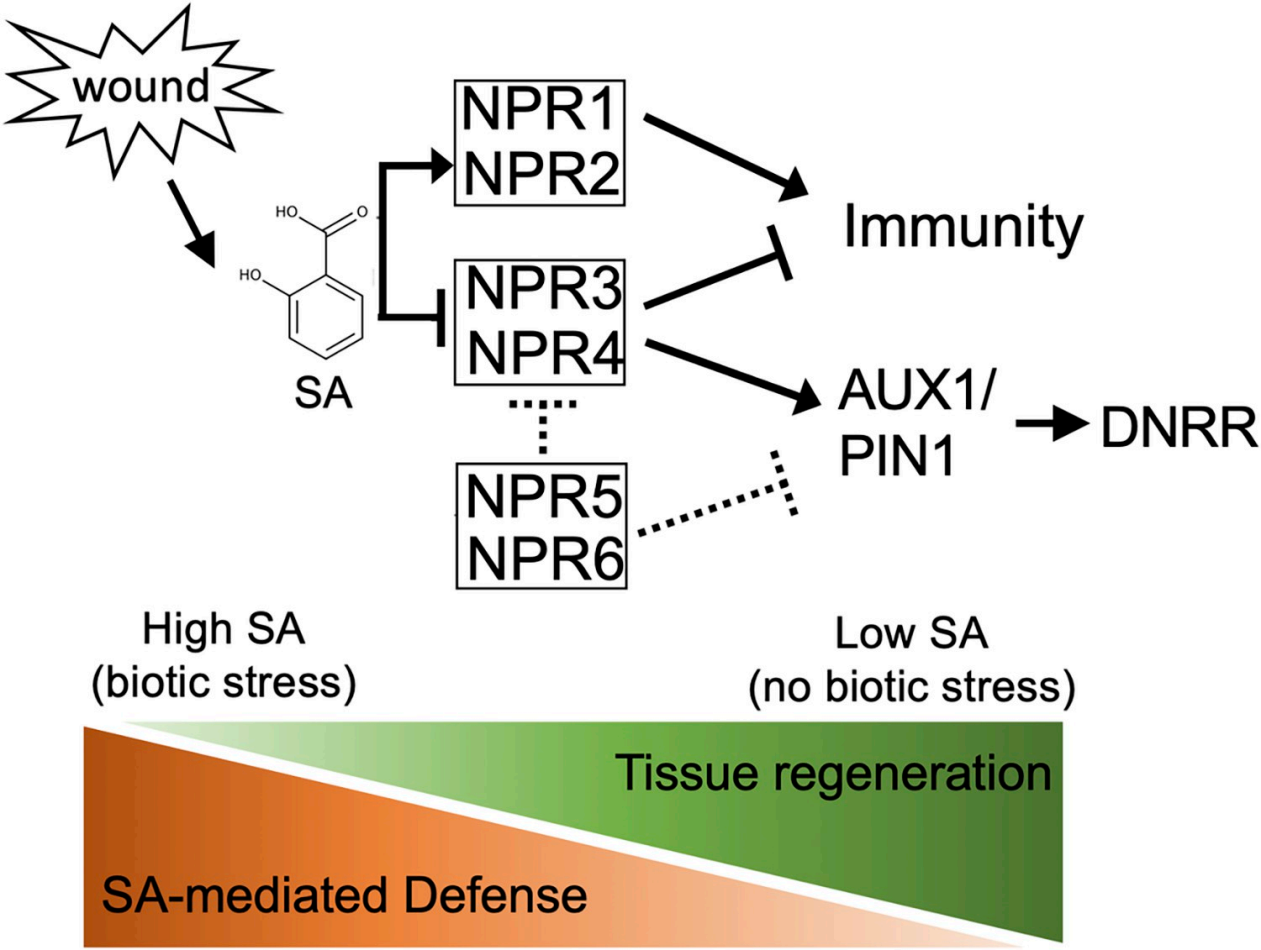
A model of SA-mediated balance of regeneration and immunity. A model depicting the role of SA signaling in DNRR. Wound-induced SA response acts through NPR3/4 to suppress the root regeneration. NPR5/6 plays an opposite role to NPR3/4. This SA-mediated suppression of DNRR may contribute to a molecular decision of regeneration or defense upon wounding.

*De novo* organogenesis is often studied in an aseptic condition, so our knowledge about how biotic stress influences this process is limited. Our study provides a potential link between biotic stress and regeneration. In the absence of biotic stress, SA level is low, and regeneration is favored; when SA is activated (e.g. after infection), the process of regeneration is suppressed (Fig 6). Although SA is an important hormone for plant-microbe interaction, direct evidence of SA in regeneration under biotic stresses is still lacking. A regeneration system compatible with microbial pathogens is needed to dissect how SA contributes to regeneration when pathogen is present. Infection may also alter other hormones that are important for regeneration such as JA and auxin [59,60]. In particular, some bacteria can enhance regeneration by generating phytohormones [61,62]. Thus, the net output of biotic stresses on regeneration can be complicated. In summary, we find that the activation of SA response and SA accumulation quickly occurs after leaf detaching. NPR3 and NPR4, but not NPR1, contribute to the SA-mediated suppression of adventitious rooting formation. SA represses the expression of genes involved in auxin transport (e.g. *PIN1* and *AUX1*) and, eventually, interferes with the essential auxin accumulation at the cutting site for cell fate transition.

## Materials and methods

### Plant materials and growth condition

Seeds used in this study are in the Columbia-0 (Col-0) background. Arabidopsis seeds were sterilized in 70% ethanol and then plated onto ½ MS media (Murashige & Skoog Basal Medium with Vitamins, PhytoTechnology Laboratories). Plates were placed in a dark room at 4°C for 2 days before moving to a growth chamber at 24°C with continuous light. Plants in soil were placed in the dark room at 4°C for 2 days and then grown in continuous light with Sun Gro Horticulture propagation mix at 24°C.

### DNRR assay

The DNRR protocol was modified from Chen *et al* 2014 [6]. Seedlings were grown in chambers with continuous light (Percival). Each 1/2MS plate host 40–60 seedlings. The 1^st^ and 2^nd^ true leaves from 12-day-old seedlings were cut at the junction between the leaf blade and petiole and placed with the abaxial side down onto Gamborg’s B5 media (RPI Research Products International) on 60 mm plates. Each plate contained 12 mL of B5 media and 20 leaf explants. Plates were sealed with micropore surgical tapes (3M). Both explants were kept in continuous light condition. The number of adventitious roots and the ratio of rooting were counted every day under a dissecting microscope (VWR) starting from the 6^th^ day after cutting up until the 14^th^ day.

### RT-PCR

For quantitative real time PCR (qRT-PCR) analysis, RNA was extracted and purified using E.Z.N.A. Plant RNA kit (Omega Bio-tek). cDNA was synthesized using SuperScript III Reverse Transcriptase (Invitrogen). PCRs were performed on a QuantStudio 5 Real-Time PCR System (Applied Biosystems) using SYBR Green PCR Master Mix (Applied Biosystems). *TUB2* (*AT5G62690*) and *SAND* (*AT2G28390*) were used as endogenous housekeeping controls. PCR primers are listed below. AUX1-F: CGGAGACGCACTTCTCGACC; AUX1-R: GAAGAGCACCGACAGCGGAA; SAND-F: AACTCTATGCAGCATTTGATCCACT; SAND-R: TGATTGCATATCTTTATCGCCATC; TUB2-F: AGCAATACCAAGATGCAACTGCG; TUB2-R: TAACTAAATTATTCTCAGTACTCTTCC; PIN1-R: CGCAGATAAGCCTTGAGACC; PIN1-F: TGATCTCCGAGCAGTTTCCA; ASA1-R: CGACCGAGATCAACCAACAT; ASA1-F:GAACCAGCAAGAGAGGGAAG; YUC2-R:CTGCATACAATCCGCTTTCG; YUC2-F:GGAGTTGAAACGGGTAATGC.

### GUS staining

Samples were stained using the protocol described by Yang *et al* 2013 [45]. To compensate for variation in GUS activity in reporter lines, the incubation time for DR5-GUS, WOX11-GUS were 8 hours and 24 hours, respectively. T0 samples were stained on intact seedlings and detached for imaging after clearing in 70% ethanol. Leaf images were taken using a dissecting scope (VWR Stereo Zoom Trinocular Microscope) and processed using the VWR V3 MP camera and ImageJ software. Quantification of GUS staining was performed using ImageJ.

### Hormone treatment

Stocks of Naphthylphthalamic acid (NPA; 10 mM) were prepared in DMSO and diluted to the final concentration (1μM) using sterile distilled water. DMSO was added in the mock treated B5 medium as a solvent control. Explants were placed on B5 medium containing the indicated concentration of NPA immediately after cutting. Sodium salicylate or BTH was used as an SA analogue. The indicated concentration of NaSA (5μM and 25μM) in autoclaved water was sprayed onto seedlings 1 hour before cutting using sterilized sprayer. After 1 hour treatment, explants were cut and immediately placed on B5 medium containing the indicated concentration of SA with abaxial side downwards. NPA and SA were also directly added into B5 media to reach the indicated concentrations as shown in figures.

### RNA-seq analysis

RNA-seq reads derived from Arabidopsis leaf explants during DNRR were previously described and deposited at the NCBI Gene Expression Omnibus (GEO) under the accession number GSE120418 [8]. The forward reads corresponding to two replicates of detached leaves of wild-type seedlings (Col-0) were used. Samples were harvested at a time course that included leaves before culture (time 0) and at 10 min, 30 min, 1h, 2h, 4h, 12h and 24h after detachment. The quality of the reads was initially assessed with FastQC version 0.11.8 (Babraham Bioinformatics, Cambridge, UK). Trimmomatic version 0.36 [63] was used to remove adaptor-containing and low-quality sequences with parameters set at ILLUMINACLIP:TruSeq3-SE.fa:2:30:10, SLIDINGWINDOW:4:5, LEADING:5, TRAILING:5, MINLEN:151. The reads were then aligned against the TAIR10 Arabidopsis reference genome using HiSAT2 version 2.2.0 [64] using default parameters. The featureCounts function from the Sub-read package version 2.0.0 [65] was used to count reads that mapped to each one of the 27,206 nuclear protein-coding genes.

Differential expression analysis was performed with the edgeR package in R [66]. Weakly expressed genes were filtered out by removing those genes that did not achieve a minimum expression level of 1 count per million in at least five libraries. Normalization was performed using the trimmed mean of M-values method (TMM; function calcNormFactors in edgeR) [67]. The Benjamini-Hochberg method (False Discovery Rate; FDR) was used for the correction of multiple comparisons [68]. Genes with an FDR lower than or equal to 0.01 and a fold-change of at least 1.5x were considered differentially expressed in the experiment. Leaves before culture (time 0) served as the reference condition for the definition of differentially expressed genes. Hierarchical clustering analyses were performed with the ComplexHeatmap package in R [69] based on the Euclidean distance and the complete-linkage method. Gene expression values in TPM (transcript per million) were normalized to z-scores for these analyses.

### SA and auxin measurement

For SA measurement, eight biological replicates per timepoint, each with ten leaves, were collected into 200μL of prechilled metabolite extraction buffer consisting of 1:1 methanol:chloroform (v/v) supplemented with ^13^C_6-_cinnamic acid, D_5_-benzoic acid, and resorcinol as internal standards [70]. Samples were sonicated in an ice-chilled water bath for 30 min. The aqueous phase was extracted by adding 100μL of high-performance liquid chromatography (HPLC)-grade water, vortexed for 30 sec, centrifuged for 5 min, and transferred to a new tube and stored at -80°C until analysis. Free SA and SA-conjugates were detected by reverse-phase HPLC-mass spectrometry (HPLC-MS, Agilent Technologies, Wilmington, DE) as described [70]. Relative abundance was determined as peak area of each metabolite divided by the mean abundance of the internal standards, followed by a correction for differences in dry-tissue weight.

Auxin measurement was performed accordingly to Muller and Munné-Bosch (2011) with minor modifications [71]. Briefly, freeze-dried tissue powder (4–6 mg) was extracted in 200 μL of buffer (20:79:1 methanol:isopropanol:glacial acetic acid, v/v/v) containing 100 ng of D_5_-IAA (CDN isotopes, Pointe-Claire, Quebec, Canada) as the internal standard. Following centrifugation, the supernatant was transferred to a new tube and the pellet was reextracted three times with 100 μL of the same buffer. All supernatants were pooled, filtered through a 0.22 μm PTFE filter (Agilent Technologies) and analyzed on an ultra-performance liquid chromatography (Agilent 1290 Infinity II UPLC) coupled to a quadrupole time-of-flight (Agilent 6546 QTOF) tandem mass spectrometer. Extracts were resolved on a reverse phase C18 column (Zorbax Eclipse Plus, 2.1 × 50 mm, 1.8 μm, Agilent) using water with 0.05% acetic acid as mobile solvent A and acetonitrile with 0.05% acetic acid as solvent B at a flow rate of 0.5 mL/min over 3 min. Negative polarity data were acquired using MS acquisition mode with the gas temperature 250°C, nebulizer gas 40 psi and capillary voltage 3000V.

Data were processed by the Qualitative analysis module of MassHunter Workstation V10.0 (Agilent). Auxin levels were quantified using a calibration curve built with authentic IAA as described (Pan et al., 2010) [72]. The authentic standard working solutions ranging from 20–500 ng/mL were prepared in methanol containing the D_5_-IAA spike-in and analyzed as above. The calibration curve was generated using the Quantitative analysis module of MassHunter Workstation V11.0 (Agilent).

## Supporting information

S1 Table Gene list of clusters in Figs 2B, 2C, S2A and S5A. List of SA-activated (sheet 1) and SA-repressed (sheet 2) genes in Fig 2B and 2C. These genes are also DEGs in at least one time point after wounding. Sheet 3: List of key genes involved in SA and auxin pathway.
(XLSX)

S2 Table Numeric data for graphs in Figures. (XLSX)

S1 Fig Rooting ratio in response to a gradient of SA. (TIFF)

S2 Fig Expression of SA responsive genes after cutting. (A) Expression pattern of genes involved in SA signaling and biosynthesis after cutting. (B) Overlap between genes in Cluster 4, Cluster 5 and JA activated genes. The light pink circle are the genes of cluster 4 (n = 101) SA up-regulated genes. The light green circle are the genes of cluster 5 (n = 91) SA up-regulated genes. In the light blue circle are genes induced by JA (n = 933) as defined in Zhang *et al* [8]. The overlapping genes represent the sector of JA-SA crosstalk.
(TIFF)

S3 Fig The DNRR capacity of *npr1* alleles. (A) Comparing the rooting ratio of Col-0 and *npr1-3* explants at different age. Explants were cut from 12-day-old or 15-day-old Col-0 or *npr1-3* plants. Each dot represents an independent experiment with 20–30 explants. Long and short bars represent means and standard errors, respectively. (B) Rooting ratio of Col-0, Nossen and *npr1-5* (No-0). X axis indicates days after cutting. Around 60 explants were analyzed for each genotype. (C) Rooting ratio of explants from F1 seedlings of a cross between *npr1-5* (No-0) and Col-0 or *npr1-1* (Col-0). Around 60 explants were analyzed for each genotype. (D) Representative images of leaf explants from Col-0 and *npr4-4D* exposed to various concentrations of SA. (E) *npr4-4D* showed reduced sensitivity to SA-mediated suppression of rooting compared to Col- 0. SA was sprayed onto seedlings 1 hour before cut and added into B5 media at the indicated concentration in A and B.
(TIFF)

S4 Fig Activation of *WOX11* in *cpr1* and the impact of NPA. (A) Activation of *WOX11* was compromised in *cpr1*. Samples of whole leaf explants were harvested at 0 and 2 DAC. *TUB2* (AT5G62690) was used as an endogenous control in qPCR analysis. Error bars indicate standard deviation of three technical repeats. (B) Rooting ratio of explants from Col-0 exposed to a gradient of NPA. (C) Rooting ratio of explants from Col-0 and NahG exposed to a gradient of NPA.
(TIFF)

S5 Fig Expression level of *ARF* genes and auxin accumulation. (A) Expression pattern of *ARF*s after cutting. (B) Accumulation of auxin at 0 DAC in Col-0, NahG and *yuc1D*. *YUC1* is overexpressed in the yuc-1D dominant mutant. Each dot represents auxin level in about 80 explants harvested from 12-day-old seedlings.
(TIFF)
