# Supplementary figures and images for: Endogenous salicylic acid suppresses de novo root regeneration from leaf explants
Source: PLoS Genet. 2023 Mar 1;19(3):e1010636. doi: 10.1371/journal.pgen.1010636 (PMC10010561; doi:10.1371/journal.pgen.1010636)

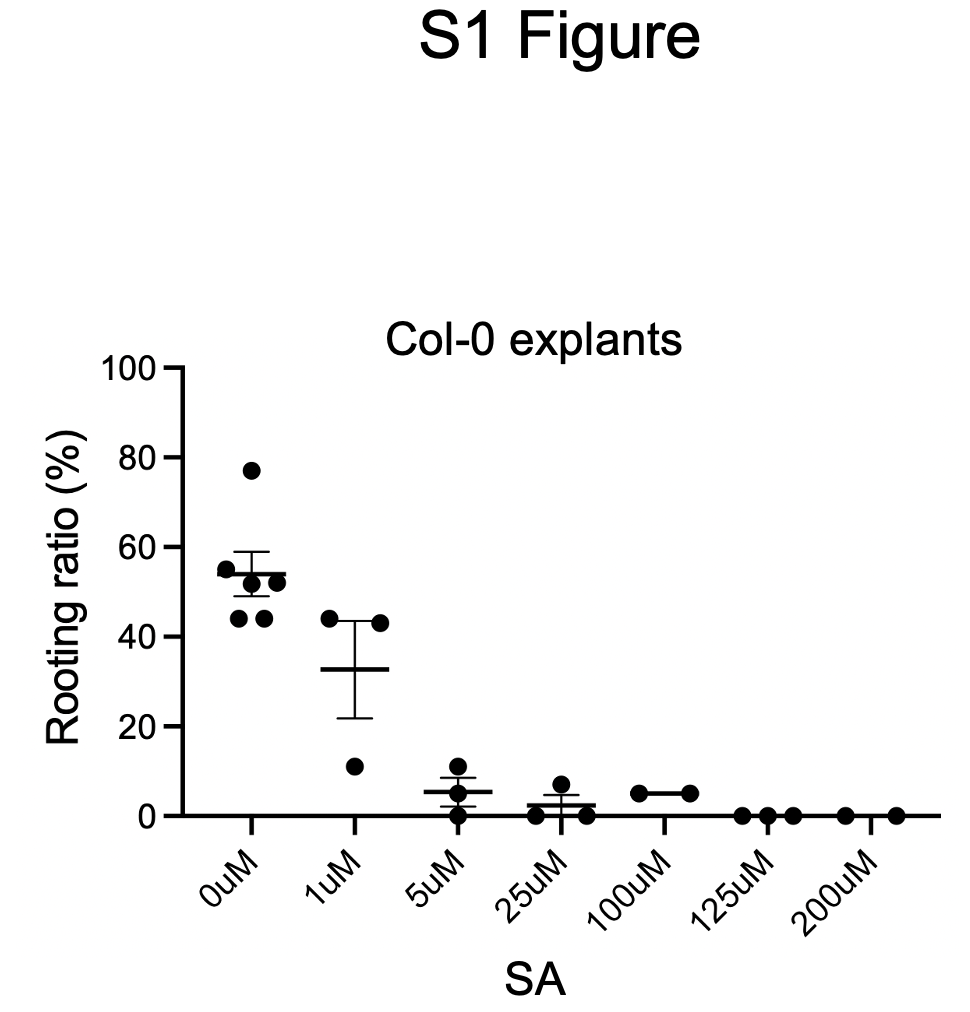

Supplement: S1 Fig — (TIFF) [file pgen.1010636.s003.tiff]

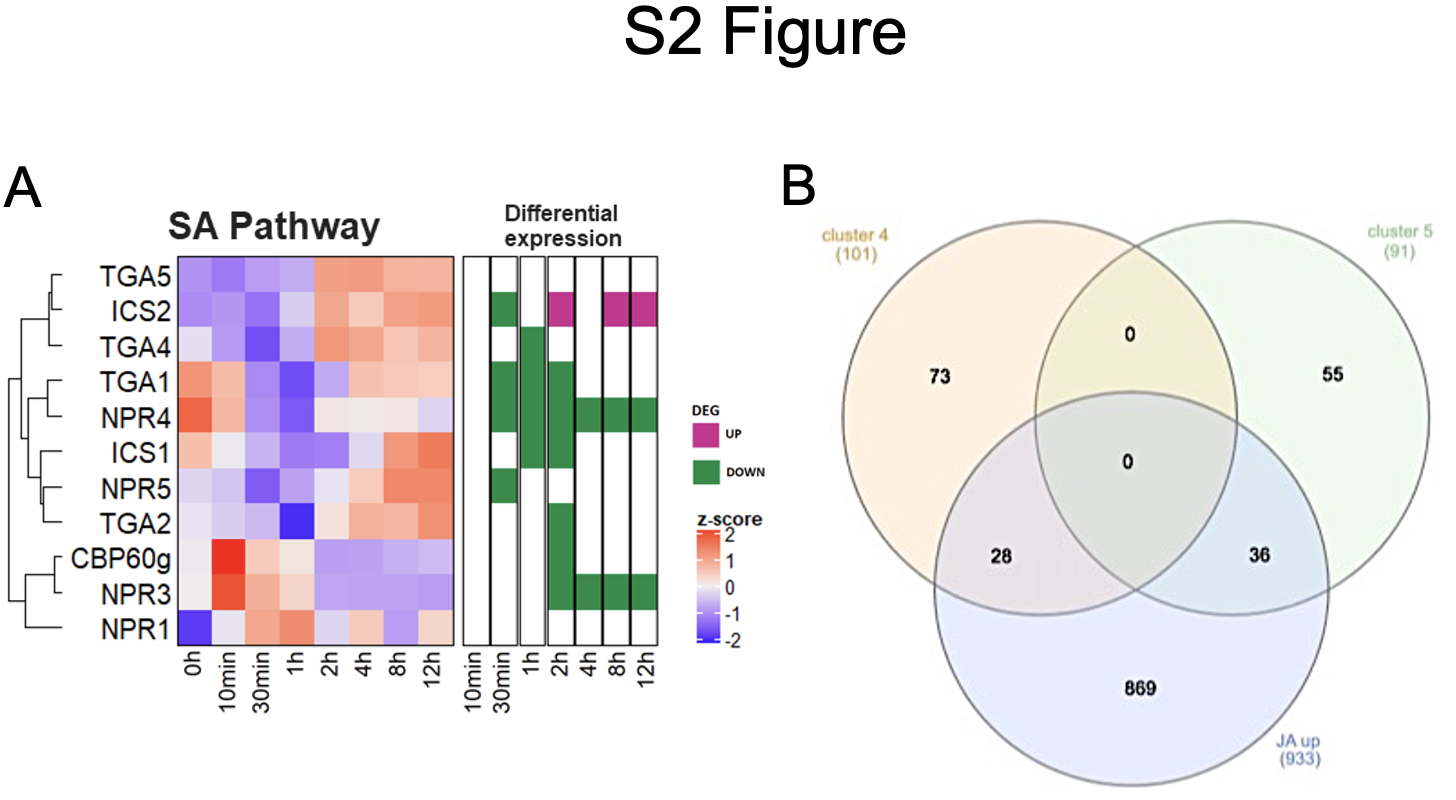

Supplement: S2 Fig — (A) Expression pattern of genes involved in SA signaling and biosynthesis after cutting. (B) Overlap between genes in Cluster 4, Cluster 5 and JA activated genes. The light pink circle are the genes of cluster 4 (n = 101) SA up-regulated genes. The light green circle are the genes of cluster 5 (n = 91) SA up-regulated genes. In the light blue circle are genes induced by JA (n = 933) as defined in Zhang et al [8]. The overlapping genes represent the sector of JA-SA crosstalk. (TIFF) [file pgen.1010636.s004.tiff]

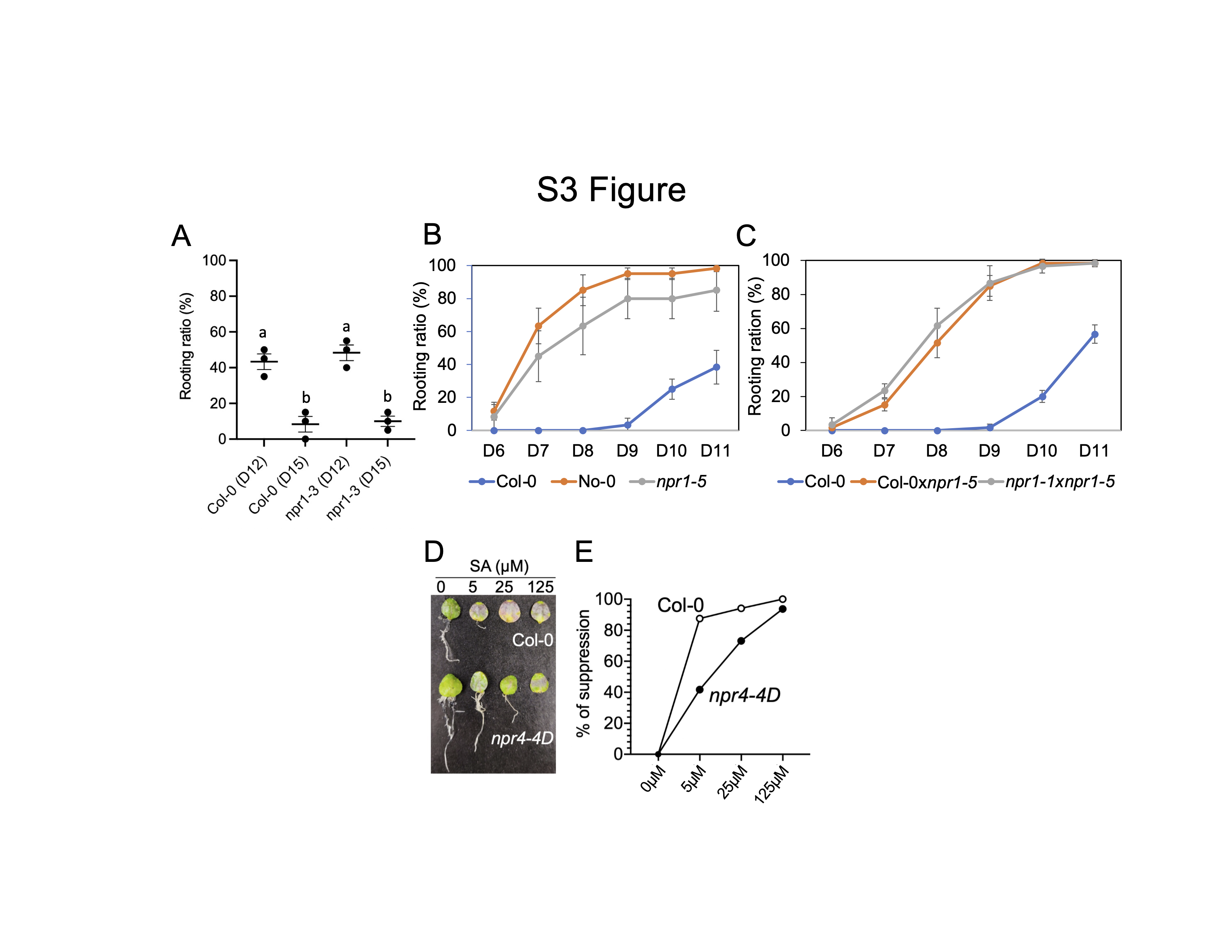

Supplement: S3 Fig — (A) Comparing the rooting ratio of Col-0 and npr1-3 explants at different age. Explants were cut from 12-day-old or 15-day-old Col-0 or npr1-3 plants. Each dot represents an independent experiment with 20–30 explants. Long and short bars represent means and standard errors, respectively. (B) Rooting ratio of Col-0, Nossen and npr1-5 (No-0). X axis indicates days after cutting. Around 60 explants were analyzed for each genotype. (C) Rooting ratio of explants from F1 seedlings of a cross between npr1-5 (No-0) and Col-0 or npr1-1 (Col-0). Around 60 explants were analyzed for each genotype. (D) Representative images of leaf explants from Col-0 and npr4-4D exposed to various concentrations of SA. (E) npr4-4D showed reduced sensitivity to SA-mediated suppression of rooting compared to Col- 0. SA was sprayed onto seedlings 1 hour before cut and added into B5 media at the indicated concentration in A and B. (TIFF) [file pgen.1010636.s005.tiff]

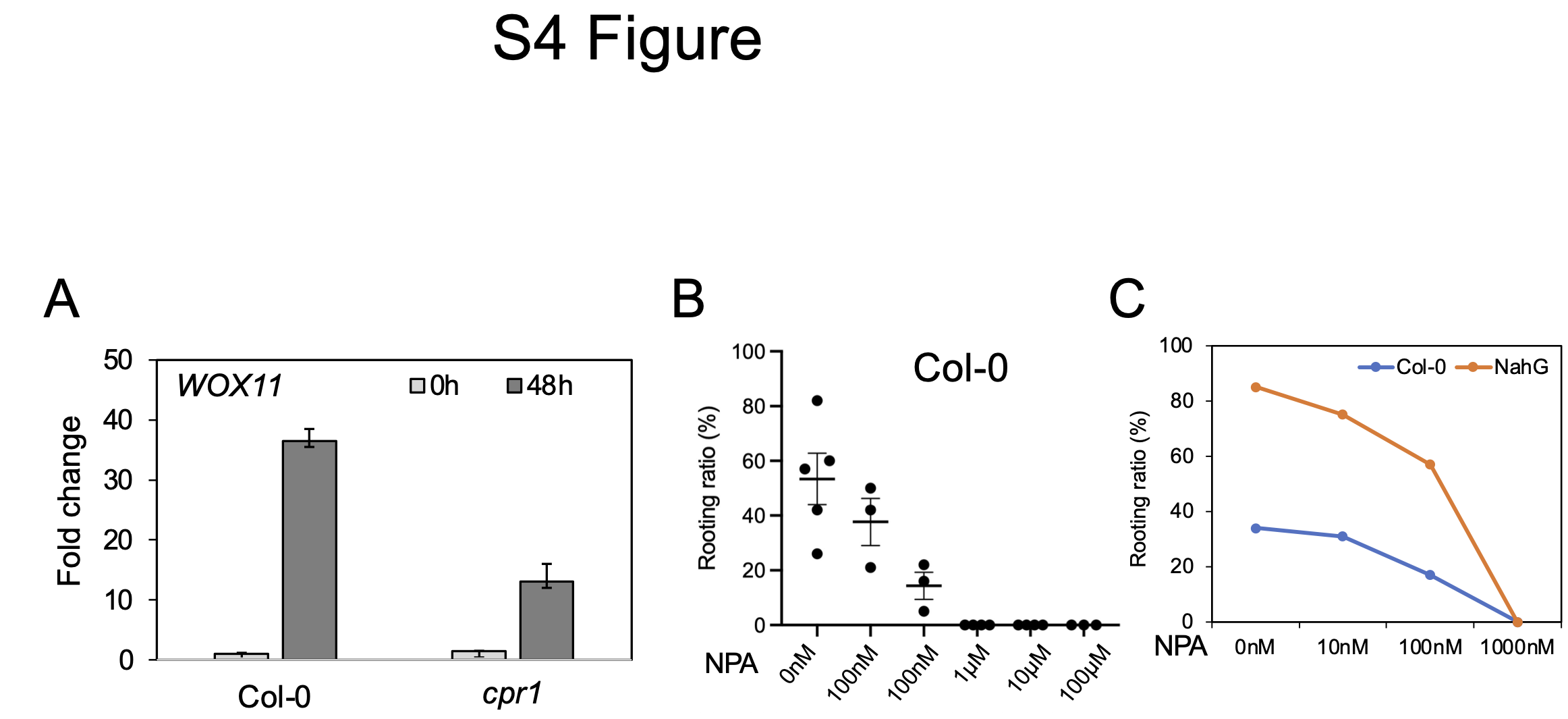

Supplement: S4 Fig — (A) Activation of WOX11 was compromised in cpr1. Samples of whole leaf explants were harvested at 0 and 2 DAC. TUB2 (AT5G62690) was used as an endogenous control in qPCR analysis. Error bars indicate standard deviation of three technical repeats. (B) Rooting ratio of explants from Col-0 exposed to a gradient of NPA. (C) Rooting ratio of explants from Col-0 and NahG exposed to a gradient of NPA. (TIFF) [file pgen.1010636.s006.tiff]

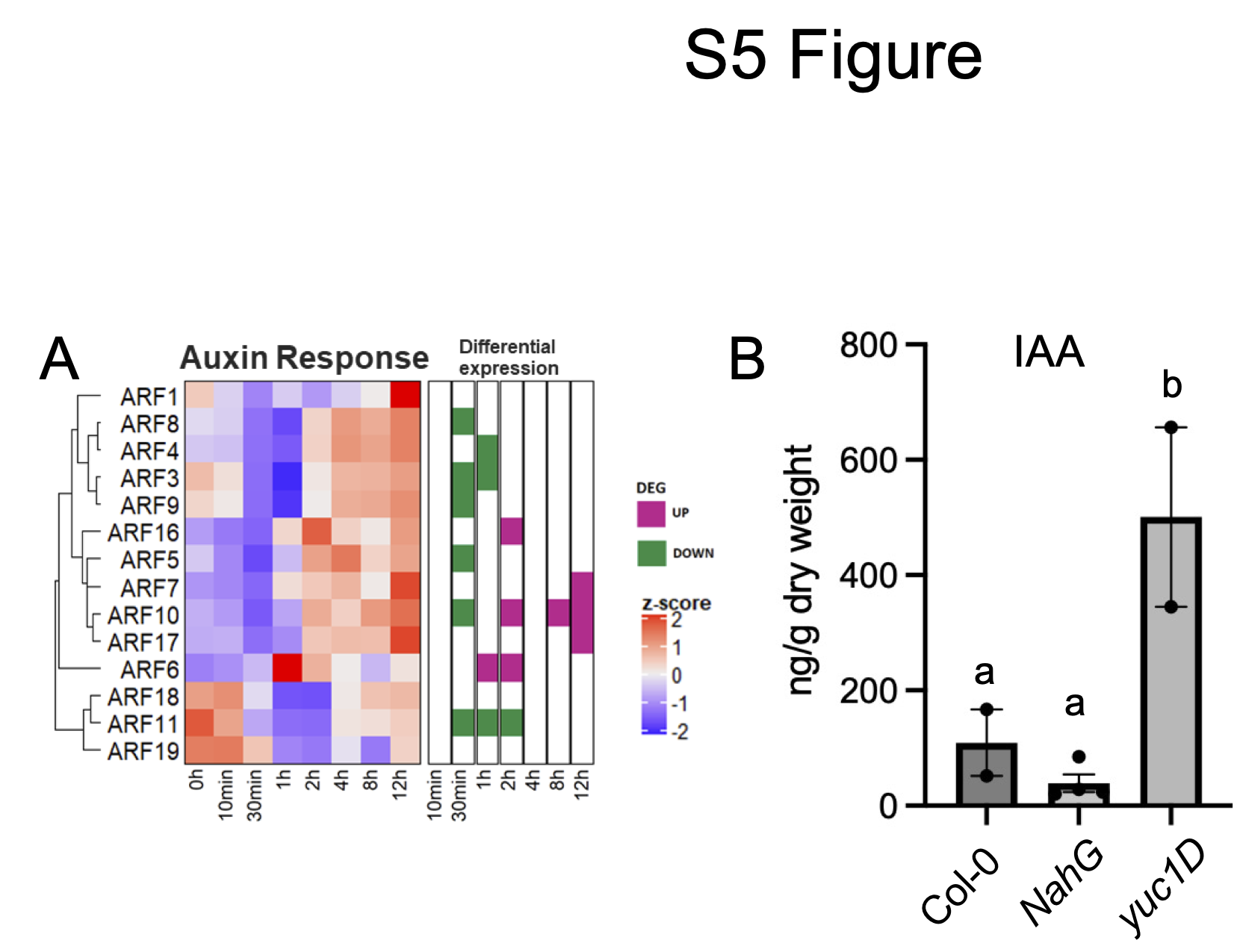

Supplement: S5 Fig — (A) Expression pattern of ARFs after cutting. (B) Accumulation of auxin at 0 DAC in Col-0, NahG and yuc1D. YUC1 is overexpressed in the yuc-1D dominant mutant. Each dot represents auxin level in about 80 explants harvested from 12-day-old seedlings. (TIFF) [file pgen.1010636.s007.tiff]
